# Antenatal care in Mozambique: Number of visits and gestational age at the beginning of antenatal care*

**DOI:** 10.1590/1518-8345.4964.3481

**Published:** 2021-10-29

**Authors:** Belarmina Reis-Muleva, Luciane Simões Duarte, Carla Marins Silva, Luciana Magnoni Reberte Gouveia, Ana Luiza Vilela Borges

**Affiliations:** 1 Universidade de São Paulo, Escola de Enfermagem, São Paulo, SP, Brazil.; 2 Bolsista da Universidade Lúrio, Nampula, Moçambique.; 3 Secretaria de Estado da Saúde de São Paulo, Coordenadoria de Controle de Doenças, Divisão de Doenças Crônicas Não Transmissíveis, São Paulo, SP, Brazil.

**Keywords:** Prenatal Care, Maternal Health, Women’s Health, Nursing Care, Gestational Age, Mozambique, Atención Prenatal, Salud Materna, Salud de la Mujer, Atención de Enfermería, Edad Gestacional, Mozambique, Assistência Pré-Natal, Saúde Materna, Saúde da Mulher, Assistência de Enfermagem, Idade Gestacional, Moçambique

## Abstract

**Objective:**

1)to assess the gestational age at the beginning of antenatal care and its covariates; 2)to assess the number of antenatal visits and its covariates; and 3)to identify the reasons for the late initiation of antenatal care and for attending less than four visits among postpartum women living in Nampula, Mozambique.

**Method:**

cross-sectional study conducted with 393 mothers who answered a structured instrument in face-to-face interviews. Logistic regression was used to analyze the covariates of having initiated antenatal care up to the 16^th^gestational week, having attended four or more antenatal visits, and reporting both situations simultaneously.

**Results:**

all postpartum women underwent antenatal care, but only 39.9% started it until the 16^th^gestational week, 49.1% attended four or more visits, and 34.1% reported both events. Having concluded high school (ORadj=1.99; 95%CI=1.19-3.31) or college (ORadj=3.87; 95%CI=1.47-10.18) were aspects associated with reporting both situations. The reasons for the late initiation of antenatal care and attending less than four visits were as follows: not finding it important to attend several visits, not having easy access to the health facility, not being aware about pregnancy, and not having a companion for the visits.

**Conclusion:**

the gestational age at the beginning of antenatal care and the number of antenatal visits are lower than the current recommendations in the country.

## Abstract (es)

**Objetivo:**

1) analizar la edad gestacional al inicio del control prenatal y aspectos asociados; 2) analizar el número de consultas realizadas y aspectos asociados; y 3) identificar las razones del inicio tardío del control prenatal y de la realización de menos de cuatro consultas por parte de las mujeres posparto que residen en Nampula, Mozambique.

**Método:**

estudio transversal con 393 puérperas, que respondieron a un instrumento estructurado en entrevistas presenciales. Se utilizó regresión logística para analizar aspectos asociados a haber iniciado el control prenatal como máximo en la semana 16 de gestación, haber asistido a cuatro o más consultas prenatales y reportar ambas situaciones simultáneamente.

**Resultados:**

todas las puérperas realizaron el control prenatal, pero solo el 39,9% lo inició como máximo en la semana 16 de gestación; el 49,1% realizó cuatro o más consultas y el 34,1% informó ambos eventos. La educación secundaria (ORaj=1,99; IC95%=1,19-3,31) o la educación superior (ORaj=3,87; IC95% 1,47-10,18) fueron aspectos asociados al informe de ambas situaciones. Las razones del inicio tardío del control prenatal y de la realización de menos de cuatro consultas fueron: no considerar que fuera importante realizar varias consultas, no tener fácil acceso al centro de salud, no saber que estaba embarazada y no tener acompañante para las consultas.

**Conclusión:**

la edad gestacional para iniciar el control prenatal y el número de consultas realizadas son inferiores a las recomendaciones vigentes en el país.

## Abstract (pt)

**Objetivo:**

1) analisar a idade gestacional de início do pré-natal e aspectos associados; 2) analisar o número de consultas realizadas e aspectos associados; e 3) identificar as razões para o início tardio do pré-natal e para a realização de menos de quatro consultas entre puérperas residentes em Nampula, Moçambique.

**Método:**

estudo transversal com 393 puérperas, que responderam a um instrumento estruturado em entrevistas face a face. Utilizou-se regressão logística para analisar aspectos associados a ter iniciado o pré-natal até 16ª semana de gestação, ter realizado quatro ou mais consultas de pré-natal e relatar as duas situações simultaneamente.

**Resultados:**

todas as puérperas realizaram pré-natal, mas apenas 39,9% iniciaram pré-natal até a 16ª semana de gravidez; 49,1% realizaram quatro ou mais consultas e 34,1% relataram ambos eventos. O ensino secundário (ORaj=1,99; IC95%=1,19-3,31;) ou superior (ORaj=3,87; IC95% 1,47-10,18) foram aspectos associados a reportar ambas situações. As razões para início tardio do pré-natal e realização de menos de quatro consultas foram: não achar importante realizar várias consultas, não ter fácil acesso ao centro de saúde, não saber que estava grávida e não ter acompanhante para as consultas.

**Conclusão:**

a idade gestacional de início do pré-natal e o número de consultas realizadas são inferiores às recomendações vigentes no país.

## Introduction

Antenatal care consists of a set of procedures and measures aimed at diagnosing, treating and preventing situations that are undesirable for women’s health - during pregnancy, delivery, postpartum - and also for the infant^(1)^. Currently, antenatal care is recognized as an important strategy to prevent or reduce the risk of maternal and newborn morbidity and mortality. The literature around this issue show that adequate antenatal care is closely linked to better perinatal outcomes and to a reduction in maternal and neonatal morbidity and mortality^(1-4)^.

Antenatal care encompasses various guidelines, recommendations, measures and procedures that, in some way, differ across countries. Even so, there are two fundamental elements that are the basis for good quality antenatal care: its early initiation and a minimum number of visits^(1,5)^.

The vast majority of high- and middle-income countries recommend at least six antenatal visits; they also recommend initiating antenatal care between the 8^th^and 12^th^gestational week^(6-7)^. However, some low-income countries have different recommendations regarding the number of visits, which at least four; and endorse the gestational age at which antenatal care is initiated with greater flexibility, up to the 16^th^week^(8-10)^. Although there are differences in the recommendations in various contexts, the World Health Organization(WHO) recommends at least eight antenatal visits, with gestational age up to the 12^th^week at its initiation^(1,5)^. It is noteworthy that these recommendations can be adapted to the socioeconomic context and to the population and health system of each country.

This is what the Ministry of Health(MISAU) of Mozambique did, a country located in Sub-Saharan Africa and scenario of this study, which recommends a minimum number of four antenatal care visits and gestational age up to the 16^th^week at its initiation^(11-12)^. In that country, few studies have assessed gestational age at the beginning of antenatal care and the number of visits^(13-14)^. A national survey showed that approximately 90% of the pregnant women in Mozambique undergo antenatal care, but only 55% attend the minimum number of four visits^(12)^. Apparently, among other reasons, late initiation is due to the guidelines provided by the health professionals themselves, who inform women that antenatal care should be started when fetal movements are felt or when the baby can be palpated; another reason is the experience of multiparous women who start antenatal care late as they already know the guidelines of the health facilities^(14)^.

Therefore, it seems that access to antenatal care in Mozambique is not necessarily the main problem in the field of reproductive health, but quality of care. The country has a high maternal mortality rate, 452*per* 100,000 live births^(15)^, which reveals that there is a need to improve the quality of maternal health care, and antenatal care does not seem to be an exception. Given the importance of antenatal care for the promotion of maternal health, especially in a country with high rates of maternal deaths, this study has the following objectives: 1)to assess the gestational age at the beginning of antenatal care and its covariates; 2)to assess the number of antenatal visits and its covariates; and 3)to identify the reasons for the late initiation of antenatal care and for reporting less than four antenatal care visits among postpartum women living in Nampula, Mozambique.

## Method

### Study design

This is a quantitative, cross-sectional and analytical study, guided by the STROBE(Strengthening the Reporting of Observational Studies in Epidemiology) tool, developed to assess the quality of observational studies(case-control, cohort and cross-sectional)^(16)^.

### Context

The study was conducted in the city of Nampula, located in the inland of the province with the same name, in northern Mozambique, Africa. According to the Human Development Report of the United Nations Development Program(UNDP), in 2015 Mozambique was among the nine countries in the world with the lowest Human Development Index(HDI), ranking in the 180^th^place out of a total of 188^(17)^. Data from the population census conducted in 2017 indicate that there are almost 28 million inhabitants, with nearly 67% of the population living in rural areas^(15)^. The mean life expectancy is 56.5 years old^(15)^ and the total fertility rate is 5.3 children *per* woman^(12)^.

### Period

The research was conducted from August to December 2019.

### Population

The study population consisted of postpartum women aged between 18 and 49 years old.

### Selection criteria

The inclusion criteria were all women whose delivery had taken place within the previous 24 hours, in maternity hospitals, and women whose delivery had taken place within the previous 15 days, at their own homes, as long as they reported being physically and emotionally willing to answer the questionnaire. The exclusion criterion was having antenatal care classified as high risk.

### Sample definition

The sample size considered the proportion of Mozambican women with four or more antenatal visits:p=54.6^(12)^, female population of Nampula of 337,839 women at the time of the study^(15)^, significance level of 95% and 5% margin of error. Sample calculation indicated the need to interview 381 women.

### Study variables

The dependent variables were gestational age at the beginning of antenatal care(up to the 16^th^week and after the 16^th^week), number of antenatal care visits(up to three and four or more) and access to basic antenatal care, herein defined as the one in which women initiated antenatal care up to the 16^th^gestational week and reported four or more antenatal care visits(dichotomous).

The independent variables referring to the sociodemographic characteristics were age group(18-24; 25-29; ≥30 years-old); schooling in years: elementary education(≤7), high school education(8-12) or higher education(>12); religion(Islamic, Catholic or others); place of residence: peripheral region(neighborhoods of Marrere, Murrapaniua, Mutauanha, Mutavarex, Memória, Muatala, Natikiri, Namicopo, Namutequeliua/Nampaco, Carrupeia) or urban region(neighborhoods of Napipine, Muhala/Muhala expansion, Muahivire, BairroCentral); paid jobs(no or yes); use of private healthcare services(no or yes); and living with a partner(no or yes). Regarding reproductive history, the number of pregnancies, including the current one(1-3 or ≥4) was assessed, as well as antenatal care in previous pregnancies(first pregnancy, in some pregnancies or in all pregnancies). As for the planning of the last pregnancy, the instrument called London Measure of Unplanned Pregnancy(LMUP) was used, validated for the Portuguese language^(18)^. Regarding the antenatal care, we assessed antenatal care visits(no or yes); the reasons for late initiation of antenatal care[multiple choice: I didn’t know I was pregnant, I didn’t think early antenatal care was important, I didn’t have anyone to accompany me(partner), care was time-consuming, the healthcare facility was far or difficult to access, difficulties related to work/school(lack of time to go to the visits), I didn’t have anyone to leave my children with, I was sick and couldn’t go to the hospital, I found my belly small, and I didn’t want this pregnancy, among others]; and the reasons for attending less than four visits[multiple choice: I didn’t want this pregnancy, I didn’t have anyone to accompany me(partner), I didn’t have anyone to leave my children with, care was time-consuming, I didn’t think it was important to attend many antenatal visits, the health center was far or difficult to access, I didn’t like the service professionals and transportation difficulties, among others].

### Instruments used to collect the information

The instrument used for data collection consisted of a specific form, prepared by the main researcher, which contained questions about the dependent and independent variables, in addition to the LMUP. LMUP is an instrument that measures pregnancy planning retrospectively, regardless of the outcome of pregnancy, birth or abortion. The classification regarding the planning of the last pregnancy is obtained by the sum of points for each question; the total score can vary from zero to 12. Women who scored between 0 and 3 points are classified as having an unplanned pregnancy; between 4 and 9 points, as ambivalent, that is, simultaneous prevalence of apparently contradictory feelings and actions about pregnancy planning and non-planning; and between 10 and 12 points as having a planned pregnancy.

### Data collection

Data collection took place in a maternity hospital and at the women’s residence. Selection of the women took place through information from the puerperium registry book available at the Health Sciences College/UniLúrio and from the maternity hospital, where information on all births that took place in the city is available. Women were interviewed using a structured instrument created in GoogleForms, pre-tested and applied using a tablet, respecting their privacy. The questionnaire included information regarding sociodemographic characteristics, reproductive history, planning of the last pregnancy and antenatal care.

### Data treatment and analysis

Analyses were performed using the Stata 15.0 software. To describe the sociodemographic characteristics, reproductive history and antenatal care, absolute and relative numbers, mean, median and standard deviation were used. The test of difference between two proportions(Chi-Square or Fisher’s Exact) was applied to verify differences between gestational age at the beginning of antenatal care, number of visits and access to basic antenatal care, and the sociodemographic characteristics, women’s reproductive history, and planning of the last pregnancy. The analysis of the covariates was performed using univariate and multiple logistic regression. In the multiple logistic regression analysis, the variables were simultaneously inserted into the model. A5% significance level was considered statistically significant.

### Ethical aspects

The project was approved by the Institutional Bioethics Committee for Health of the Lúrio University(*Comitê Institucional de Bioética para Saúde da Universidade Lúrio*,CIBSUL) of Nampula, Mozambique, under No.26/August/CIBSUL/19.

## Results

A total of 416 women were invited to the take part in the study, but 17 refused to participate. Another six women were not found at their homes to conduct the interview after three attempts; thus, the final sample consisted of 393 women. The mean age was 26.0 years old(SD=5.8), most had elementary education(52.4%), lived in the peripheral region of the city(75.3%), used public health system(91.1%) and lived with a partner(88.8%). Nearly one out of 10 women had a paid job(10.9%). Almost half reported professing the Islamic religion(43.4%), number of pregnancies greater than or equal to four(44.5%) and had their pregnancies classified as planned(41.0%) (Table 1).

**Table 1:** Number and proportion of women, according to sociodemographic characteristics, reproductive history and planning of the last pregnancy. Nampula, Nampula, Mozambique, 2019

Characteristics	N	%
Age group (years old)		
Mean (SD)	26.0 (5.8)	
18-24	170	43.3
25-29	103	26.2
≥30	120	30.5
Schooling (years)		
Elementary school (≤7)	206	52.4
High school (8-12)	152	38.7
Higher education (>12)	35	8.9
Religion*		
Islamic	169	43.4
Catholic	137	35.2
Others	83	21.4
Place of residence		
Peripheral region	296	75.3
Urban region	97	24.7
Paid job		
No	350	89.1
Yes	43	10.9
Uses the private healthcare system		
No	358	91.1
Yes	35	8.9
Lives with a partner		
No	44	11.2
Yes	349	88.8
Number of pregnancies, including the current one		
Mean (SD)	3.3 (1.7)	
1-3	218	55.5
≥4	175	44.5
Antenatal care in previous pregnancies^†^		
Primiparous	88	22.6
In some pregnancies	44	11.3
In all pregnancies	257	66.1
Planning of the last pregnancy		
Not planned	122	31.0
Ambivalent	110	28.0
Planned	161	41.0
**Total**	**393**	**100.0**

* 4 women were excluded for not reporting any religion;

† 4 women were excluded for not undergoing antenatal care in previous pregnancies

All women reported having undergone antenatal care in their last pregnancy(which is being considered in this study), and 39.9%(n=157) started antenatal care up to the 16^th^gestational week; 49.1%(n=193) reported at least four antenatal visits, and 34.1%(n=134) were categorized as having undergone basic antenatal care, that is, they started antenatal care up to the 16^th^gestational week and had at least four antenatal visits(data not shown in the table).

Among women who started antenatal care after the 16^th^gestational week(n=236,60.1%), the main reasons were thinking that the belly was small(42.6%), not having a spouse as a companion for the visits(24.1%) and not knowing that they were pregnant(19.8%), among others. Among women who reported less than four antenatal visits(n=200,50.9%), the main reasons were not finding it important to have several antenatal visits(36.0%), not having a spouse as a companion for the visits(25.5%) and not having easy access to the health facility or living far from the health service(24.5%), among others(Table 2).

**Table 2:** Number and proportion of women according to reasons for late initiation of antenatal care and for attending less than four visits. Nampula, Nampula, Mozambique, 2019

Reasons	n	%
**Reasons for starting antenatal care after the 16^th^ gestational week (n=236)**		
Considering the belly small	101	42.6
Not having a spouse as a companion for the visits	57	24.1
Not being aware about the pregnancy	47	19.8
Not finding it important to start antenatal care early	38	16.0
Not wanting the pregnancy	36	15.2
Not having easy access to the health facility or living far from the health service	24	10.1
Being tired/sick and not being able to go to the health facility	18	7.6
Not having someone to leave the other children with	12	5.1
Having difficulty in undergoing antenatal care due to work/school	6	2.5
Reporting delayed service in the health center	4	1.7
Reporting neglect regarding their own health care	4	1.7
Reporting insecurity and fear of not reaching the end of the pregnancy and of being vaccinated in the hospital	2	0.8
Reporting lack of knowledge about the period of the first visit	1	0.4
Reporting the nurses’ requirement for the presence of the partner in the visits	1	0.4
**Reasons for attending less than four antenatal visits (n=200)**		
Not finding it important to attend several antenatal visits	72	36.0
Not having a spouse as a companion for the visits	57	25.5
Not having easy access to the health facility or living far from the health service	49	24.5
Not wanting the pregnancy	38	19.0
Reporting difficulty in transportation to the health facility	33	16.5
Reporting delayed service in the health facility	19	9.5
Not having someone to leave the other children with	16	8.0
Reporting not liking the health professionals	7	3.5
Considering the belly small	4	2.0
Being tired/sick and not being able to go to the health center	3	1.5
Reporting being tired of attending or walking to the visits	2	1.0
Reporting fear of being vaccinated at the hospital	2	1.0
Reporting neglect in the care of the child, as they have many children	1	0.5
Having been hospitalized for a long time	1	0.5
Waiting to get through the first six months of pregnancy	1	0.5
Not being able to walk because of swollen feet	1	0.5
Not being aware about the pregnancy	1	0.5
Reporting deceased spouse	1	0.5
Reporting the partner’s prohibition to attend antenatal visits	1	0.5

The analysis of the aspects associated with the initiation of antenatal care up to the 16^th^week is presented in Table 3. Women with high school education(OR=1.89; 95%CI=1.23-2.92) or higher education(OR=4.25; 95%CI=1.99-9.07) were more likely to start antenatal care up to the 16^th^week, when compared to those with elementary education. Women who reported having used the private healthcare system(OR=2.45; 95%CI=1.20-4.98) were more likely to start antenatal care up to the 16^th^week, when compared to those who used the public healthcare system, as was also the case of the women with paid job(OR=2.56; 95%CI=1.34-4.89). In the multiple model, adjusted for all the variables entered simultaneously, only education remained associated with the initiation of antenatal care up to the 16^th^week, considering both women with high school education(ORadj=1.66; 95%CI=1.02-2.69) and with higher education(ORadj=2.70; 95%CI=1.05-6.96), when compared to those with elementary education.

**Table 3:** Aspects associated with the initiation of antenatal care up to the 16^th^gestational week. Nampula, Nampula, Mozambique, 2019

Characteristics	Initiation of antenatal care up to the 16^th^ week		Univariate analysis		Multiple analysis*	
	N	%	OR	95%CI	ORadj^†^	95%CI
Age group (years old)	0.910					
18-24	69	40.6	1.00	-	1.00	-
25-29	42	40.8	1.00	0.61-1.66	1.07	0.58-1.99
≥30	46	38.3	0.91	0.56-1.47	1.08	0.51-2.27
Education (years)	<0.001					
Elementary school (≤7)	64	31.1	1.00	-	1.00	-
High school (8-12)	70	46.0	1.89	1.23-2.92	1.66	1.02-2.69
Higher education (>12)	23	65.7	4.25	1.99-9.07	2.70	1.05-6.96
Religion^‡^	0.249					
Islamic	63	37.3	1.00	-	1.00	-
Catholic	63	46.0	1.43	0.91-2.27	1.30	0.80-2.13
Others	31	37.3	1.00	0.58-1.73	0.97	0.54-1.73
Place of residence	0.210					
Peripheral region	113	38.2	1.00	-	1.00	-
Urban region	44	45.4	1.34	0.85-2.14	1.04	0.63-1.73
Lives with a partner	0.135					
No	13	29.6	1.00	-	1.00	-
Yes	144	41.3	1.67	0.85-3.31	1.77	0.83-3.75
Uses the private healthcare system	0.011					
No	136	38.0	1.00	-	1.00	-
Yes	21	60.0	2.45	1.20-4.98	1.32	0.58-3.00
Paid job	0.004					
No	131	37.4	1.00	-	1.00	-
Yes	26	60.5	2.56	1.34-4.89	1.50	0.70-3.21
Number of pregnancies, including the current one	0.152					
1-3	94	43.1	1.00	-	1.00	-
≥4	63	36.0	0.74	0.49-1.12	0.72	0.39-1.36
Antenatal care in previous pregnancies^§^	0.403					
Primiparous	40	45.4	1.00	-	1.00	-
In some pregnancies	19	43.2	1.25	0.66-2.40	1.14	0.57-2.30
In all pregnancies	97	37.7	1.37	0.84-2.24	1.26	0.69-2.33
Pregnancy planning	0.105					
Not planned	46	37.7	1.00	-	1.00	-
Ambivalent	37	33.6	0.84	0.49-1.43	0.80	0.45-1.40
Planned	74	46.0	1.41	0.87-2.27	1.19	0.71-2.00

* Hosmer and Lemeshow test:p=0.3785;

† Adjusted *Odds Ratio*;

‡ 4 women were excluded for not reporting any religion;

§ 4 women were excluded for not undergoing antenatal care in previous pregnancies

The association between women’s characteristics and reporting at least four antenatal care visits is shown in Table 4. Women who were more likely to report at least four antenatal visits were those with high school education(OR=2.50; 95%CI=1.63-3.85) or higher education(OR=19.43; 95%CI=5.75-65.65), who lived with a partner(OR= 2.87; 95%CI=1.43-5.76), who used the private healthcare system(OR=2.83; 95%CI=1.32-6.06), and who had paid job(OR=9.56; 95%CI=3.68-24.87). Among these characteristics, in the adjusted model, women with high school education(ORadj=2.12; 95%CI=1.29-3.46) or higher education(ORadj=11.78; 95%CI=2.91-47.71), who lived with a partner(ORadj= 3.11; 95%CI=1.39-6.92), and who reported paid job (ORadj=4.80; 95%CI=1.65-13.96) were more likely to report at least four antenatal care visits.

**Table 4:** Aspects associated with attending at least four antenatal care visits. Nampula, Nampula, Mozambique, 2019

Characteristics	Four or more visits		Univariate analysis		Multiple analysis*	
	n	%	OR	95%CI	ORadj^†^	95%CI
Age group (years old)	0.830					
18-24	81	47.7	1.00	-	1.00	-
25-29	53	51.5	1.16	0.71-1.90	1.69	0.87-3.29
≥30	59	49.2	1.06	0.66-1.70	1.89	0.85-4.21
Schooling (years)	<0.001					
Elementary school (≤7)	73	35.4	1.00	-	1.00	-
High school (8-12)	88	57.9	2.50	1.63-3.85	2.12	1.29-3.46
Higher education (>12)	32	91.4	19.43	5.75-65.65	11.78	2.91-47.71
Religion^‡^	0.121					
Islamic	79	46.8	1.00	-	1.00	-
Catholic	77	56.2	1.46	0.93-2.30	1.40	0.83-2.34
Others	36	43.4	0.88	0.51-1.48	0.72	0.39-1.33
Place of residence	0.085					
Peripheral region	138	46.6	1.00	-	1.00	-
Urban region	55	56.7	1.50	0.94-2.38	0.91	0.53-1.58
Lives with a partner	0.002					
No	12	27.3	1.00	-	1.00	-
Yes	181	51.9	2.87	1.43-5.76	3.11	1.39-6.92
Uses the private health system	0.006					
No	168	46.9	1.00	-	1.00	-
Yes	25	71.4	2.83	1.32-6.06	0.85	0.31-2.29
Paid work	<0.001					
No	155	44.3	1.00	-	1.00	-
Yes	38	88.4	9.56	3.68-24.87	4.80	1.65-13.96
Number of pregnancies, including the current one	0.069					
1-3	116	53.2	1.00	-	1.00	-
≥ 4	77	44.0	0.69	0.46-1.03	0.49	0.25-0.96
Antenatal care in previous pregnancies^§^	0.260					
Primiparous	50	56.8	1.00	-	1.00	-
In some pregnancies	22	50.0	1.14	0.60-2.16	0.92	0.42-1.98
In all pregnancies	120	46.7	1.50	0.92-2.45	1.68	0.88-3.19
Pregnancy planning	0.049					
Not planned	58	47.5	1.00	-	1.00	-
Ambivalent	45	40.9	0.76	0.45-1.29	0.54	0.30-0.97
Planned	90	55.9	1.40	0.87-2.24	0.97	0.56-1.66

* Hosmer and Lemeshow test:p=0.2508;

† Adjusted *Odds Ratio*;

‡ 4 women were excluded for not reporting any religion;

§ 4 women were excluded for not undergoing antenatal care in previous pregnancies

The analysis of the aspects associated with having accessed basic antenatal care is shown in Table 5. Women who were more likely to access basic antenatal care were those with high school education(OR=2.33; 95%CI=1.48-3.68) or higher education(OR=6.31; 95%CI=2.92-13.61), who were Catholics(OR=1.74; 95%CI=1.08-2.80), who used the private healthcare system(OR=2.51; 95%CI=1.24-5.06) and who had paid job(OR=3.43; 95%CI=1.79-6.58). In the final model, only education remained statistically significant. Thus, women with high school education(ORadj=1.99; 95%CI=1.19-3.31;) or higher education(ORadj=3.87; 95%CI=1.47-10.18) were more likely to access basic antenatal care.

**Table 5:** Aspects associated with access to basic antenatal care. Nampula, Nampula, Mozambique, 2019

Characteristics	Basic antenatal care		Univariate analysis		Multiple analysis*	
	n	%	OR	95%CI	ORadj^†^	95%CI
Age group (years old)	0.971					
18-24	59	34.7	1.00	-	1.00	-
25-29	35	34.0	0.97	0.57-1.62	1.16	0.60-2.24
≥30	40	33.3	0.94	0.57-1.54	1.27	0.57-2.83
Schooling (years)	<0.001					
Elementary school (≤7)	48	23.3	1.00	-	1.00	-
High school (8-12)	63	41.4	2.33	1.48-3.68	1.99	1.19-3.31
Higher education (>12)	23	65.7	6.31	2.92-13.61	3.87	1.47-10.18
Religion^‡^	0.069					
Islamic	49	29.0	1.00	-	1.00	-
Catholic	57	41.6	1.74	1.08-2.80	1.67	0.99-2.80
Others	28	33.7	1.25	0.71-2.19	1.18	0.64-2.17
Place of residence	0.144					
Peripheral region	95	32.1	1.00	-	1.00	-
Urban region	39	40.2	1.42	0.89-2.28	1.00	0.59-1.69
Lives with a partner	0.091					
No	10	22.7	1.00	-	1.00	-
Yes	124	35.5	1.87	0.90-3.92	1.98	0.88-4.45
Uses the private healthcare system	0.008					
No	115	32.1	1.00	-	1.00	-
Yes	19	54.3	2.51	1.24-5.06	1.07	0.46-2.51
Paid job	<0.001					
No	108	30.9	1.00	-	1.00	-
Yes	26	60.5	3.43	1.79-6.58	1.89	0.87-4.09
Number of pregnancies, including the current one	0.153					
1-3	81	37.2	1.00	-	1.00	-
≥4	53	30.3	0.73	0.48-1.12	0.73	0.37-1.43
Antenatal care in previous pregnancies^§^	0.117					
Primiparous	38	43.2	1.00	-	1.00	-
In some pregnancies	16	36.4	1.26	0.65-2.47	1.07	0.51-2.26
In all pregnancies	80	31.1	1.68	1.02-2.76	1.76	0.93-3.31
Pregnancy planning	0.073					
Not planned	41	33.6	1.00	-	1.00	-
Ambivalent	29	26.4	0.71	0.40-1.25	0.62	0.34-1.14
Planned	64	39.8	1.30	0.80-2.13	1.01	0.59-1.73

* Hosmer and Lemeshow test:p=0.3922;

† Adjusted *Odds Ratio*;

‡ 4 women were excluded for not reporting any religion;

§ 4 women were excluded for not undergoing antenatal care in previous pregnancies

## Discussion

This study aimed at assessing both the gestational age at the beginning of antenatal care and the number of antenatal care visits and their associated aspects; as well as identifying the reasons for the late initiation of antenatal care and less than four visits, according to the MISAU recommendations^(11)^. Antenatal care coverage was found to be universal in the municipality of Nampula, Mozambique. However, the quality of prenatal care was still low, as less than half of women started antenatal care early and attended the minimum number of recommended visits. When these two indicators are analyzed together, the condition of access to basic antenatal care seemed even more fragile.

Our findings are similar to the few studies already conducted in Mozambique on the topic. In a qualitative study conducted in the provinces of Cabo Delgado, Tete and Maputo, which represented the three regions of the country(North, Central and South), it was observed that, in general, women started antenatal care at the end of the second trimester, and that the reasons for this late initiation were lack of a pregnancy test in the health services and waiting for the presence of visible signs of pregnancy^(13)^. Similar findings were identified in another study conducted in five Mozambican provinces, in which 55% of pregnant women started antenatal care between 20 and 28 weeks of gestational age^(14)^. Therefore, it seems that little progress has been made on Mozambican context regarding women`s ability of making full use of antenatal care, starting it early and accessing a greater number of contacts with health professionals.

Such results were also observed in other countries that adopted similar antenatal quality parameters: 9%to 48%variation in the adequacy of gestational age at the beginning of antenatal care and 27% to 45% in the number of visits, considering other African countries, as well as countries in the American and Asian continents^(3,8,19)^. It is noteworthy that, despite the different political, economic and social contexts across these countries, the results are similar. Although our findings cannot be directly compared with other studies that assess the quality of antenatal care, as we analyzed only two indicators and some other studies often consider other indicators, the access to adequate antenatal carein Mozambique was observed in 45% of the pregnancies elsewhere^(13)^. That said, our results confirm that are still limitations in the health services regarding the early recruitment of pregnant women in the country.

Considering that early initiation of antenatal care and more visits allow for more qualified care and better maternal and fetal results^(1,5)^, the reasons for its late initiation and lower number of recommended visits were also investigated in this study. Considering the women’s reports, the reasons that stood out the most were the fact that they did not think it was important to attend several visits; did not have easy access to the health facility; found their belly small; did not know that they were pregnant, and did not have a companion for the visits. Similar data were found in studies carried out in Mozambique^(13-14)^ and in other regions of the world^(10,19)^. This is a very worrying finding, as it shows the influence of beliefs and values on access to and continuity of antenatal care in the city of Nampula^(20)^. In addition to promoting access to rapid pregnancy diagnosis methods, which allow for earlier recruitment of the pregnant woman, to encouraging the partner’s participation in antenatal visits, to supporting women who do not want their pregnancy, and to reframing strategies that facilitate access to the healthcare facilities, it is fundamental to demystify certain cultural aspects related to pregnancy.

Furthermore, a systematic review study on the perception of the importance of antenatal care and barriers to access in 15 low-income countries, including Mozambique^(10)^, showed that women perceive pregnancy as a normal life event, that is, as a physiological event in which antenatal care is only necessary in situations of disease. Thus, little understanding of the benefits of antenatal care and a preference for popular care methods are observed. In addition to that, antenatal care visits demand long wait at the healthcare services, and this time could be used for household chores and for taking care of other children; in addition to the fact that the inadequate transportation structure in some regions of Mozambique represents exposure to risks for the pregnancy^(10)^. Our results confirm the findings of the aforementioned review.

Women with more years of schooling were more likely to start antenatal care early, report the appropriate number of visits and report both situations simultaneously, which is widely documented in several other studies^(8-9,21-26)^. Education enables women to develop the confidence needed to make decisions about their own body and health and to more easily understand the importance of the antenatal care services; in addition to the fact that a higher schooling level is generally related to greater autonomy in the decision to seek antenatal care. Other studies have also shown that women with more years of schooling are the ones who most perform preconception preparation and who experience less unplanned pregnancies^(27-28)^, events that are markedly related to better levels of maternal health. Therefore, investment in women’s education will benefit women and their fetuses.

Having a paid job was associated with attending four or more antenatal visits which is similar to other studies^(8,24)^. It is understood that women with paid job are often those with greater autonomy in the decision to seek antenatal care and better schooling. In addition to that, paid job enables economic independence so that woman can pay for the costs of transportation to the healthcare service, which enables access to healthcare.

Additionally, women who lived with their partners were more likely to attend four or more visits when compared to those who did not live with a partner, a fact that is similar to results obtained in studies carried out in Brazil^(29-30)^. Thus, the absence of a partner can constitute a potential weakness for accessing antenatal care. This may be because the presence of a partner encourages the performance of high quality antenatal care, since the presence of men in antenatal visits offers well-being to women and, somehow, encourages adherence to the treatment and to preventive measures^(1,5)^.

On the other hand, the lack of support from an intimate partner or from family members leaves women more fragile and vulnerable and, in turn, negatively affects the search for adequate antenatal care. Culturally, pregnancies outside marriage can be considered disapproved and considered a shameful act in many African societies, and this may discourage single or unaccompanied women from seeking antenatal visits^(31)^. Social support from family members and/or friends can be strengthening for pregnant women who are not in a stable relationship.

Although we have only considered variables related to the women’s individual characteristics in our analysis of the covariates of the initiation of antenatal care up to the 16^th^gestational week and reporting a minimum of four antenatal care visits, it is necessary to highlight that the responsibility for improving the access and offering high-quality antenatal care lies in the MISAU, as well as in the healthcare services themselves, which need to respond to the health needs of pregnant women or those planning to become pregnant.

To our knowledge, this is the first study in Nampula, Mozambique, which assessed the covariates of gestational age at the beginning of antenatal care, the number of visits, and the simultaneity of these two events. Another potential of our study lies in the fact that we used a validated instrument to assess pregnancy planning. Knowing the profile of women with access to so-called basic antenatal care contributes to the proposal of public health and social policies that effectively improve women’s health, as well as that of their future children. Thus, it is necessary to think of strategies so that women with low schooling and without any paid job may, through the public healthcare service, diagnose pregnancy early and access information about the importance of starting antenatal care early and, consequently, attend more antenatal care visits.

Health professionals, especially nurses and maternal nurses, common in Mozambique, can also act to demystify beliefs and values, such as finding the belly small and not considering it important to attend antenatal care; they can also contribute to expanding access to reproductive planning, as some women reported not knowing that they were pregnant and not knowing how to take care of a future pregnancy.

In addition to that, Mozambique adopts a system of fewer antenatal care visits than the number recommended by the WHO. It is noteworthy that countries that adopt a reduced number of antenatal visits present a reduction in the associated cost without changes in the maternal mortality rates, with the aggravating factor of an increase in perinatal mortality and lower women’s satisfaction^(32)^.

This study has some limitations. In the first place, women living in a single municipality and attending the Mozambican National Healthcare System were evaluated; therefore, the findings cannot be generalized to Mozambican women in general, nor to those who use the private healthcare system. However, considering that antenatal care coverage should be universal, results of research implemented around the quality of public health services may help to improve the quality of antenatal care for every women, irrespective if attending public or private services. In the second place, our data were obtained from the women’s reports and memory bias can have occurred, as some women may have omitted or forgotten some information during the interviews. To minimize this bias, interviews were done with postpartum women(24-hour period, among women who gave birth in the maternity ward, and up to 14 days postpartum, among women who gave birth at their homes).

Our study also contributes by identifying the difficulties of the antenatal care services in a country in Sub-Saharan Africa, which may support the implementation of initiatives aimed at increasing the number of visits and anticipating the initiation of antenatal care, such as increasing the schooling of children, adolescents and women; offering support so that they can reach the health facility without major difficulties; and providing information so that pregnant women understand the importance of antenatal care. Such initiatives are not only necessary in African countries, but also in other low- and middle-income countries that still struggle to achieve the best possible levels of maternal health.

## Conclusion

Our findings showed that, despite the universal coverage of antenatal care, the gestational age at the beginning of antenatal care and the number of visits are lower than the current recommendations in Mozambique. For the social and public health policy-makers, improvements are suggested in the access to health care facilities, in the early recruitment of pregnant women, in the demystification of beliefs for adherence to antenatal care, and in the provision of care on reproductive planning to support women who plan to become pregnant.
